# Case Report: Tocilizumab for Acute Kidney Graft Dysfunction in Patient Affected by COVID-19

**DOI:** 10.3389/fmed.2021.732792

**Published:** 2021-11-25

**Authors:** Infante Barbara, Mercuri Silvia, Troise Dario, Castellano Giuseppe, Giovanni Stallone

**Affiliations:** ^1^Nephrology Dialysis and Transplantation Unit, University of Foggia, Foggia, Italy; ^2^Unit of Nephrology, Dialysis and Renal Transplantation—Fondazione Istituto di Ricovero e Cura a Carattere Scientifico (IRCCS) Ca'Granda Ospedale Maggiore Policlinico di Milano, Milan, Italy; ^3^Department of Clinical Sciences and Community Health, University of Milan, Milan, Italy

**Keywords:** COVID-19, tocilizumab, graft dysfunction, acute kidney injury, transplantation

## Abstract

Severe acute respiratory syndrome coronavirus 2 (SARS-CoV-2) was isolated in January 2020 and, on March, the WHO declared the status of a pandemic. It causes a cytokine release syndrome, called “cytokine storm,” characterized by systemic inflammation involving elevated levels of cytokines and hyperactivation of immune cell; this profound alteration in the immune system led to an overshooting inflammatory response contributing to morbidity and mortality. Solid organ transplant recipients are at particularly higher risk of developing critical coronavirus disease 2019 (COVID-19) due to chronic immunosuppression; in fact, establishing the balance between infection and rejection in any transplant recipient is the principal aim when prescribing immunosuppression. Tocilizumab, a humanized monoclonal antibody against interleukin-6 (IL-6) receptor widely adopted in adult rheumatoid arthritis, is used as rescue therapy for chronic antibody-mediated rejection in kidney transplantation. Data about the use of tocilizumab for treating acute kidney graft dysfunction in a setting of kidney-transplanted patients affected by COVID-19 are lacking. In this case study, we discuss the case of kidney transplant recipient with proven SARS-CoV-2 infection that develops acute graft dysfunction and the management of immunosuppression with concomitant tocilizumab administration.

## Introduction

Severe acute respiratory syndrome coronavirus 2 (SARS-CoV-2) is an RNA virus, belonging to the β-coronavirus family, causing an influenza-like syndrome potentially deadly. SARS-CoV-2 was isolated in January 2020 and, on March, the WHO declared the status of pandemic (1). Viral transmission occurs mainly through direct contact, droplets, or during procedures such as tracheal intubation and bronchoscopy. When the virus enters into the organism, it binds to a receptor named angiotensin-converting enzyme 2 (ACE2), found in many organs including the lung, kidney, liver, heart, and intestine (2). SARS-COV-2 causes a cytokine release syndrome, called “cytokine storm,” characterized by a systemic inflammation involving elevated levels of cytokines and immune cell hyperactivation (2), leading to further tissue injury. A significant increase has been extensively demonstrated in the plasma concentration of interleukin-1 (IL-1), IL-1 receptor antagonist (IL-1RA), interleukin-6 (IL-6), interleukin-7 (IL-7), interleukin-8 (IL-8), interleukin-10 (IL-10), interferon-gamma (IFN-G), monocyte chemoattractant protein (MCP)-1, granulocyte-colony stimulating factor (G-CSF), macrophage inflammatory protein (MIP)-1A, MIP-1B, and tumor necrosis factor-alpha. Moreover, the severity of lung injury was strongly associated with increased several cytokines. A dysregulated immune system, resulting in an overshooting inflammatory response, contributes to morbidity and mortality (3, 4).

Coronavirus disease 2019 (COVID-19) pandemic has led to more than 140 million SARS-CoV-2-infected patients and more than 3 million deaths (5). Surprisingly, in solid organ transplant (SOT) recipients, a relatively small number of patients has been reported in case reports or in small case series (6).

Theoretically, SOT recipients are at particularly higher risk of developing critical COVID-19 due to chronic immunosuppression. The presence of comorbid conditions is associated with higher risk of death, which is concerning because significant comorbidity is common in recipients of transplants. The additional risk posed by immunosuppression in these patients cannot be estimated due to lack of data. For these, clinicians should extrapolate from evidence obtained from treating other viral infections in transplants recipients including the reduction or discontinuing immunosuppression. This raises the importance of maintaining immunosuppression and investigating novel methods to prevent and treat the occurrence of acute kidney graft dysfunction in this particular clinical setting. In this case study, we discuss the case of kidney transplant recipient with proven SARS-CoV-2 infection that develops an acute graft dysfunction and the management of its immunosuppressive therapy with the introduction of tocilizumab (7).

## Case Report

A 61-year-old woman underwent kidney transplantation from a deceased donor in 1995. The immunosuppressive treatment included cyclosporine 50 mg twice a day (BD) and methylprednisolone 4 mg once a day (OD). She had a history of insulin-dependent type 2 diabetes and hypertension with pacemaker implantation in 2009.

On 13th November, she communicated by telephone the onset of dyspnea, fever, and cough. On the next day, a nasopharyngeal swab for SARS-CoV-2 was performed with positive results; therefore, she was admitted to our division. Physical examination showed increased respiratory rate (35 breaths/min), oxygen saturation at 88%, while the patient was breathing ambient air. The etiology of chronic kidney disease was unknown; serum creatinine level at admission was 1 mg/dl with normal levels of serum electrolytes, transaminase, and coagulation. A chest X-ray was performed on the first day of admission showing typical radiological findings of COVID-19 pneumonia with bilateral multifocal alveolar opacities and bilateral pleural effusion. Therefore, we started oxygen supplementation through Venturi mask (12 l/min); since, the persistence of a hypoxic state, she required noninvasive ventilation by bilevel positive airway pressure (BPAP) with the improvement of oxygen saturation to 96%. Additionally, we started intravenous steroid administration (methylprednisolone 20 mg BID), cephalosporin, lysine acetylsalicylate, and diuretics (furosemide) with cyclosporine dosage reduced to 50%. No specific antiviral drugs were given; due to progressive worsening of respiratory condition (oxygen saturation was of 90%, respiratory rate > 35 breaths/min), we decided to withdrawn cyclosporine. On day 2, the patient showed oliguria (300 ml/24 h) with an increase in serum creatinine levels to 2.8 mg/dl (baseline 1 mg/dl), a high white blood cell count (12.68 × 10^9^/L) with lymphocyte percentage significantly lower (6.9%), and neutrophil percentage markedly higher (89.8%) than baseline values. Additionally, C-reactive protein (108.4 mg/l) and serum IL-6 (58.21 pg/ml) were significantly elevated.

**Figure 1:**
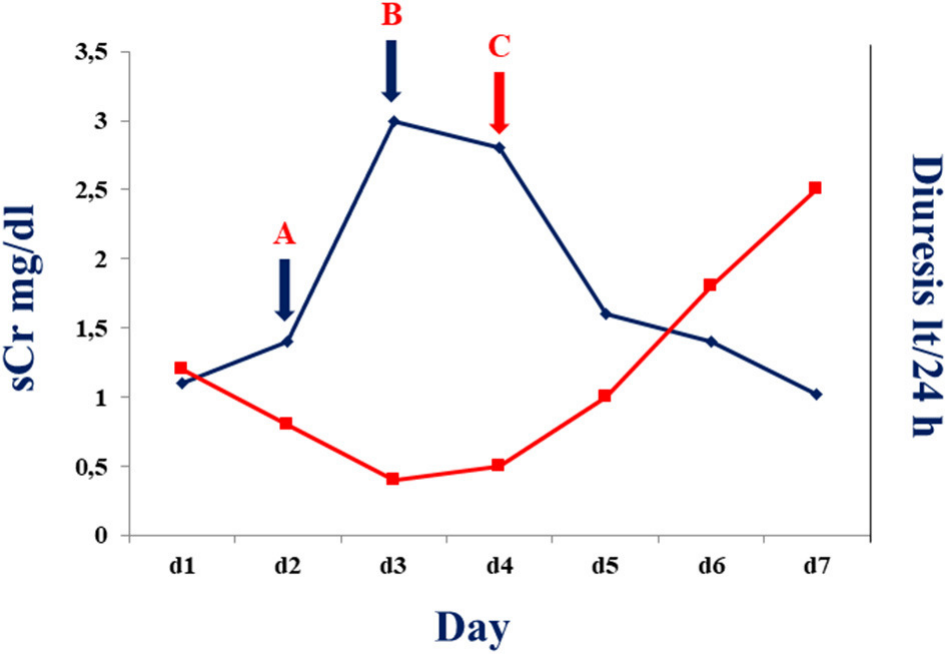
Serum creatine (blue line) and diuresis (red line) before and after tocilizumab administration in the described patient. A: Serum creatinine and diuresis at time of 50% cyclosporine dose reduction. B: Serum creatinine and diuresis at time of withdrawn cyclosporine administration. C: Serum creatinine and diuresis after 24–48 h of tocilizumab administration.

**Figure 2:**
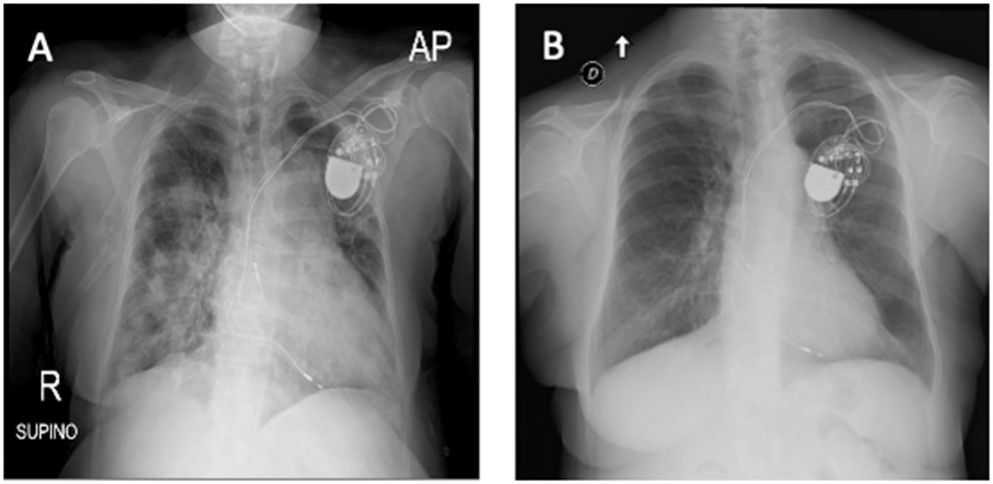
**(A)** X-ray at hospital admission describing parenchymal thickening and a bilateral widespread pulmonary interstitial involvement. **(B)** Tocilizumab administration led to bilateral improvement with a decrease in the density and in the extension of lung thicknesses (X-ray).

Considering our previous observation (7), we decide to administer an intravenous single dose of anti-IL-6 receptor monoclonal antibody (tocilizumab; 8 mg/kg; total dose of 600 mg). On the next day, the patient showed increased diuresis (about 800 ml/24 h), an oxygen saturation of 94%, and a mild improvement of tachypnea (respiratory rate < 30 breaths/min). Forty-eight hours after tocilizumab administration, white blood cell count significantly decreased (6.02 × 10^9^/L) with a rise of lymphocyte percentage (12.8%) and a mildly decrease of neutrophil percentage (83%). C-reactive protein was consistently declined to 1.6 mg/l with improvement of graft function (Figure 1). After 4 days from tocilizumab administration, physical examination revealed an improvement of the respiratory clinic with a stable oxygen saturation on the ventilator around 99%, respiratory rate of 29 breaths/min, and serum creatinine returned to baseline level (1.02 mg/ml) (Figure 1); a second X-ray was performed showing a reduction of the pulmonary opacities (Figure 2A, X-ray at hospital admission; Figure 2B, X-ray after tocilizumab administration). According to reduced oxygen requirement, noninvasive ventilation was stopped and oxygen supplementation at 15 L/min through Venturi mask was maintained until progressive reduction of the oxygen flow. We then introduced tacrolimus at low dosage (3 mg/day with trough level between 4.0 and 6.0 ng/ml) and progressively tapered methylprednisolone until 8 mg/day; interestingly, we observed a progressive increase in 24/h diuresis to 3,000 ml. The second nasopharyngeal swab for SARS-CoV-2 performed on 16th day of admission was negative and the patient was discharged on the same day.

## Discussion

In this case study, we describe, for the first time, the effect of tocilizumab administration for acute graft dysfunction in a kidney-transplanted patient affected by acute respiratory distress syndrome (ARDS) due to COVID-19.

Tocilizumab, a humanized monoclonal antibody against IL-6 receptor widely adopted in adult rheumatoid arthritis, is used as rescue therapy for chronic antibody-mediated rejection in kidney transplantation (8); tocilizumab has been registered for treating severe or life-threatening chimeric antigen receptor T-cell-induced cytokine release syndrome in adult and pediatric patients (9). In this case study, due to the development of ARDS in COVID-19 pneumonia has been associated with activation of the immune system and consequent cytokine storm with high levels of IL-6, some initial reports suggested a beneficial role of this drug (10) also in solid organ transplanted patients (6).

Interleukin-6 is a multifunctional cytokine that promotes T-cell growth, activation and differentiation of B cells, and regulates the acute phase response in systemic inflammation. IL-6 is an important mediator of inflammation that is critical to shaping T-cell immunity and inhibiting regulatory T (Tregs) cells, while increasing T-helper 17 (Th17) cells populations (11, 12); IL-6 is also critical for the progression of naïve B cells to mature plasma cells (12). Excessive IL-6 production has been linked to several human diseases characterized by unregulated antibody production and autoimmunity (13). Reports have shown that tocilizumab also reduces antibody-producing cells, diminishes inflammatory markers, and improves clinical symptomatology in other autoimmune diseases (11, 13, 14). Other clinical observations and animal models have shown that IL-6 plays a pivotal role in mediating the allograft rejection; IL-6 production increases in mouse allografts undergoing rejection and is responsible for allogeneic T-cell infiltration (15). Additionally, IL-6 deficiency or inhibition with anti-IL-6, along with costimulatory pathway blockage by cytotoxic T-lymphocyte-associated protein 4 (CTLA4), induces graft acceptance (16). Data from relevant animal models are also supportive of an important role for IL-6 in the mediation of allograft vasculopathy (17).

All these observations induced a deep reflection about the management of immunosuppression that is a priority for the clinician to be directed at maintaining balance for preventing acute graft rejection and more severe disease manifestations correlated with COVID-19. In this case study, the data in the literature are currently controversial; several authors agree with the need to reduce immunosuppressive therapy according to the severity of the disease, since a regular immunosuppression can lead to a slow viral deletion in the infected cells. Moreover, data about the use of tocilizumab as an immunosuppressive drug for treating acute graft dysfunction are lacking. Several reports have described the use of tocilizumab administration promoting a favorable outcome in some transplant patients focused on reduced requirement of oxygen (O_2_) therapy and on improvement of lung lesions and other studies reported higher mortality in transplant patients treated with tocilizumab (18–20); however, none of these reported the effects on graft function. Alternatively, there is a paucity of data on the mechanistic and biological impact of tocilizumab on the immune system; recently, Zarinsefat et al. (21) explored the impact of tocilizumab on immune system activation through stimulated cells derived by kidney-transplanted patients with subclinical rejection enrolled in an investigator-initiated clinical trial (*NIAID U01 AI113362-01*; https://grantome.com/grant/NIH/U01-AI113362-06). Interestingly, the authors showed that many inflammation-related pathways linked to acute rejection were suppressed in cells exposed to tocilizumab. Therefore, these data shed light on the biological effects of this drug for treating acute kidney graft dysfunction, other than on COVID-19 infection cytokine release syndrome.

In conclusion, this case study underlines the importance of modulation of immunosuppressive therapy in the setting of kidney transplant patients and the use of tocilizumab can be an important option to prevent and/or to treat the occurrence of acute kidney graft dysfunction in these patients affected by SARS-COV-2 infection. We are aware that our observation does not represent a definitive conclusion and needs rigorous assessment in controlled clinical trials.

## Data Availability Statement

The original contributions presented in the study are included in the article/supplementary material, further inquiries can be directed to the corresponding author/s.

## Ethics Statement

The studies involving human participants were reviewed and approved by University of Foggia. The patients/participants provided their written informed consent to participate in this study.

## Author Contributions

IB and MS wrote the paper. TD, CG, and GS substantially revised the manuscript and critically reviewed it. All authors contributed to the article and approved the submitted version.

## Conflict of Interest

The authors declare that the research was conducted in the absence of any commercial or financial relationships that could be construed as a potential conflict of interest. The handling editor declared a past co-authorship with several of the authors CG and GS.

## Publisher's Note

All claims expressed in this article are solely those of the authors and do not necessarily represent those of their affiliated organizations, or those of the publisher, the editors and the reviewers. Any product that may be evaluated in this article, or claim that may be made by its manufacturer, is not guaranteed or endorsed by the publisher.
